# Trends in maternal and child health in China and its urban and rural areas from 1991 to 2020: a joinpoint regression model

**DOI:** 10.1038/s41598-024-63689-2

**Published:** 2024-06-12

**Authors:** Xin‒yue Wang, Bei‒bei Zhang, Yuan‒yi Cao, Qian Xue, Qin Ye, Yuan‒sheng Li, Shu‒yuan Wang, Yuan‒wei Ma, Yan‒que Sun, Jun‒hui Zhang

**Affiliations:** 1 Department of Epidemiology and Health Statistics, School of Public Health, Southwest Medical University, No.1, Section 1, Xianglin Road, Longmatan District, Luzhou, Sichuan People’s Republic of China; 2 School of Public Health, Southern Medical University, Guangzhou, 510515 Guangdong People’s Republic of China

**Keywords:** Maternal and child health, Neonatal mortality rate, Infant mortality rate, Under-five mortality rate, Maternal mortality ratio, Joinpoint regression model, Health care, Health policy, Public health

## Abstract

The long-term trends in maternal and child health (MCH) in China and the national-level factors that may be associated with these changes have been poorly explored. This study aimed to assess trends in MCH indicators nationally and separately in urban and rural areas and the impact of public policies over a 30‒year period. An ecological study was conducted using data on neonatal mortality rate (NMR), infant mortality rate (IMR), under-five mortality rate (U5MR), and maternal mortality ratio (MMR) nationally and separately in urban and rural areas in China from 1991 to 2020. Joinpoint regression models were used to estimate the annual percentage changes (APC), average annual percentage changes (AAPC) with 95% confidence intervals (CIs), and mortality differences between urban and rural areas. From 1991 to 2020, maternal and child mortalities in China gradually declined (national AAPC [95% CI]: NMRs − 7.7% [− 8.6%, − 6.8%], IMRs − 7.5% [− 8.4%, − 6.6%], U5MRs − 7.5% [− 8.5%, − 6.5%], MMRs − 5.0% [− 5.7%, − 4.4%]). However, the rate of decline nationally in child mortality slowed after 2005, and in maternal mortality after 2013. For all indicators, the decline in mortality was greater in rural areas than in urban areas. The AAPCs in rate differences between rural and urban areas were − 8.5% for NMRs, − 8.6% for IMRs, − 7.7% for U5MRs, and − 9.6% for MMRs. The AAPCs in rate ratios (rural vs. urban) were − 1.2 for NMRs, − 2.1 for IMRs, − 1.7 for U5MRs, and − 1.9 for MMRs. After 2010, urban‒rural disparity in MMR did not diminish and in NMR, IMR, and U5MR, it gradually narrowed but persisted. MCH indicators have declined at the national level as well as separately in urban and rural areas but may have reached a plateau. Urban‒rural disparities in MCH indicators have narrowed but still exist. Regular analyses of temporal trends in MCH are necessary to assess the effectiveness of measures for timely adjustments.

## Introduction

During social development, improving maternal and child health (MCH) has always been considered fundamental to promoting the health of the population and enhancing the quality of life^1^. Key MCH indicators include neonatal mortality rate (NMR), infant mortality rate (IMR), under-five mortality rate (U5MR), and maternal mortality ratio (MMR). With rapid global economic development and improvements in medical care, MCH has improved substantially worldwide. However, as of 2015, only 62 countries had successfully achieved the Millennium Development Goal 4 of reducing child mortality^2^. Currently, Africa has the highest U5MR and MMR worldwide, which are lowest in developed regions such as Western Europe and Canada and remain at intermediate levels in Asia^3^.

China is one of the countries that has achieved the Millennium Development Goals for maternal and child mortalities^4^. Before the 1990s, MCH indicators in China reflected poor women and children's health, with significant inequality between urban and rural areas^5,6^. To improve MCH, Chinese government formulated a series of policies and goals, such as the "Healthy China 2030" plan (2016)^7^, the China National Program for Women's Development (2021–2030), and the China National Program for Child Development (2021–2030)^8^, which place higher demands for MCH than set by the United Nations. Many studies found the temporal trends of MCH indicators in China have a remarkable improvement over the past few decades^9,10^, and the disparity between urban and rural areas has narrowed considerably, although it persists. To further reduce the urban‒rural disparity, there is a need to track trends in MCH levels and the underlying reasons for these changes.

Most previous studies were based on data from the past twenty years^11^, with fewer utilizing data from the last 30 years. Existing research primarily focused on national or regional levels^10,12^, with less analysis conducted simultaneously across the national and urban‒rural dimensions. In terms of research methods, most of these studies use general descriptive analysis^13^ or classical time series models such as Grey-Markov model and autoregressive integrated moving average (ARIMA) model^14,15^. Although these methods have certain advantages in data prediction, they cannot fully capture the localized variation patterns and inflection point information of the data. In contrast, the joinpoint regression model is a segmented linear model that is capable of identifying inflection points, dividing a period into segments, and thus assisting in revealing both global and local characteristics of data changes^16^. This model has been increasingly used to evaluate the temporal trend characteristics of diseases such as cancer^17^ and infectious diseases^18^. Currently, no study has used this model to comprehensively assess the temporal trends and urban‒rural disparities of China's MCH indicators over the past 30 years at both the national and urban‒rural levels. Therefore, the present study used joinpoint regression models to examine trends in MCH indicators in China nationwide and separately in urban and rural areas from 1991 to 2020, as well as trends in urban‒rural disparities. These findings can serve as a scientific reference for MCH policy planning.

## Methods

### Data source

The data on national and urban‒rural NMR, IMR, U5MR, and MMR from 1991 to 2020 were obtained from the China Statistical Yearbook 2021^19^, published by the National Health Commission Statistical Information Center (China Statistical Yearbook 2021) (stats.gov.cn). The data for maternity and children in the Yearbook were collected through China’s National Maternal and Child Mortality Surveillance System. The surveillance system employed stratified cluster random sampling to select monitoring units, and strict reporting and quality control procedures were implemented^20^.

### Definitions

The NMR was defined as the number of deaths before 28 days of age per 1000 live births in a given year^21^. IMR was defined as the number of deaths before 1 year of age per 1000 live births in a given year^22^. U5MR was defined as the number of deaths before five years of age per 1000 live births in a given year^23^. MMR was defined as the number of maternal deaths per 100,000 live births in a given year^24^.

In China, urban areas include prefecture-level municipalities and municipalities under the direct control of the central government, and rural areas include counties and county-level cities. Data on township health centres, and village health offices are counted as rural areas^19^.

The inequality between urban and rural mortality was assessed using two indicators: rate difference (RD) and rate ratio (RR). RD is defined as rural mortality minus urban mortality, while RR is defined as the ratio of rural to urban mortality^25^. These two indicators provide a more precise reflection of the urban‒rural gap in MCH.

### Statistical analysis

Data from the China Statistical Yearbook were downloaded into Microsoft Excel 2019 (Microsoft, Redmond, WA, USA), then analysed using SPSS 26.0 (IBM, Armonk, NY, USA). Trends in the four MCH indicators across China and separately in urban and rural areas from 1991 to 2020, as well as disparities in mortality between urban and rural areas, were analysed using joinpoint regression models^16^ in the Joinpoint Regression Program 4.9.1.0 (US National Cancer Institute, https://surveillance.cancer.gov/joinpoint/). The joinpoint regression models included linear and log-linear models. Since the data for the four MCH indicators in the present study were not normally distributed, log-linear models were used. Monte Carlo permutation tests with 4499 randomly permuted datasets were applied to determine the numbers and locations of the inflection points and to estimate the regression parameters. Based on the fitted joinpoint regression model, Annual percentage changes (APC) was calculated to describe changes in the slopes within each time segment. The Average annual percentage changes (AAPC), derived by weighting segment lengths, provided a comprehensive measure of trends in MCH indicators over the entire observation period. Notably, when the number of inflection points is zero, AAPC equals APC. Positive APC indicated increasing MCH mortality during the time segment under analysis, negative APC indicated decreasing MCH mortality, and 0 indicated a stable state. The APCs and AAPCs with 95% confidence intervals (CIs) were calculated for all of China and separately for urban and rural areas of the country. The significance of trends was assessed using t-tests, with *P* < 0.05 considered statistically significant.

### Ethics approval and consent to participate

Due to the use of the IHME database, the requirement for informed consent was waived. Data on the annual NMRs, IMRs, U5MRs, and MMRs in China from 1991 to 2020 at the national level and separately in urban and rural areas are publicly available without any personal identifying information. Links to the analysed data can be found in the section on the availability of data and materials. All procedures were performed in accordance with relevant guidelines.

## Results

### Descriptive analysis of MCH indicators

**Figure 1:**
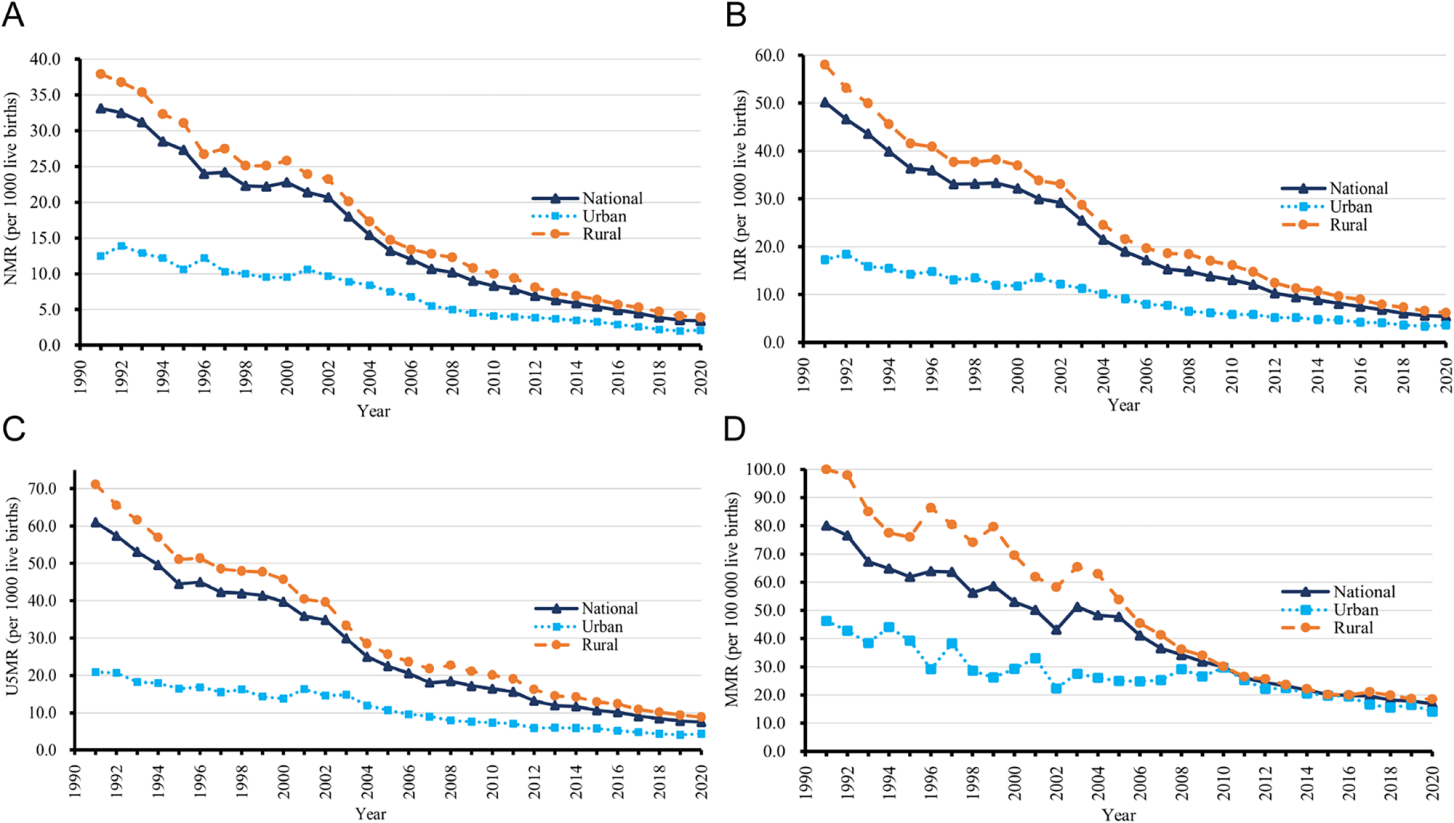
National, urban, and rural neonatal mortality rates (NMRs) (**A**), infant mortality rates (IMRs) (**B**), under-five mortality rates (U5MRs) (**C**), and maternal mortality rates (MMRs) (**D**) in China from 1991 to 2020.

From 1991 to 2020, NMRs, IMRs, U5MRs, and MMRs in China showed overall downward trends at the national level and separately in urban and rural areas (Fig. 1). Notably, the four MCH indicators were higher in rural areas than in urban areas. The downward trends in rural areas were notable and similar to the national trends, whereas the trends in urban areas were relatively moderate. Supplementary Table S1 shows the national and urban/rural NMRs, IMRs, U5MRs, and MMRs and percent changes between 1991 and 2020 in China. The percent changes in all three child health indicators were greater (≥ 87.5%) in rural areas and nationwide than in urban areas.

**Figure 2:**
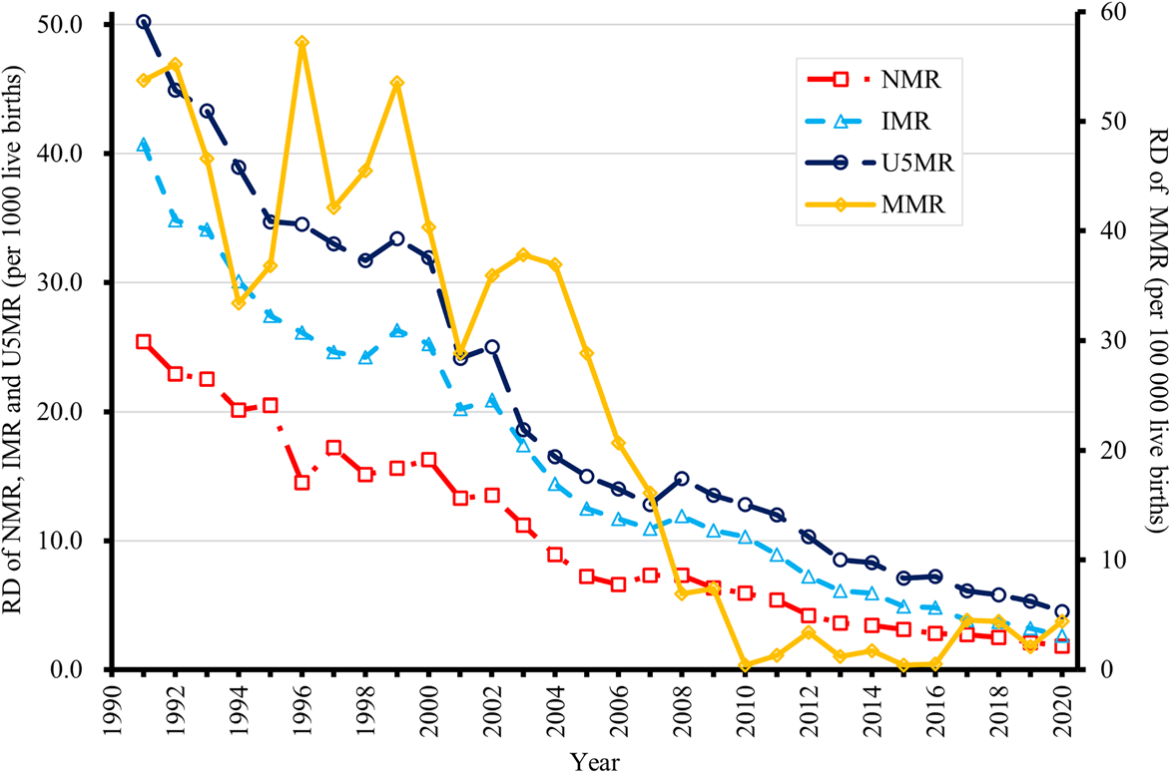
Rate differences (RDs) of national and urban/rural neonatal mortality rates (NMRs), infant mortality rates (IMRs), under-five mortality rates (U5MRs), and maternal mortality rates (MMRs) in China, 1991–2020.

**Figure 3:**
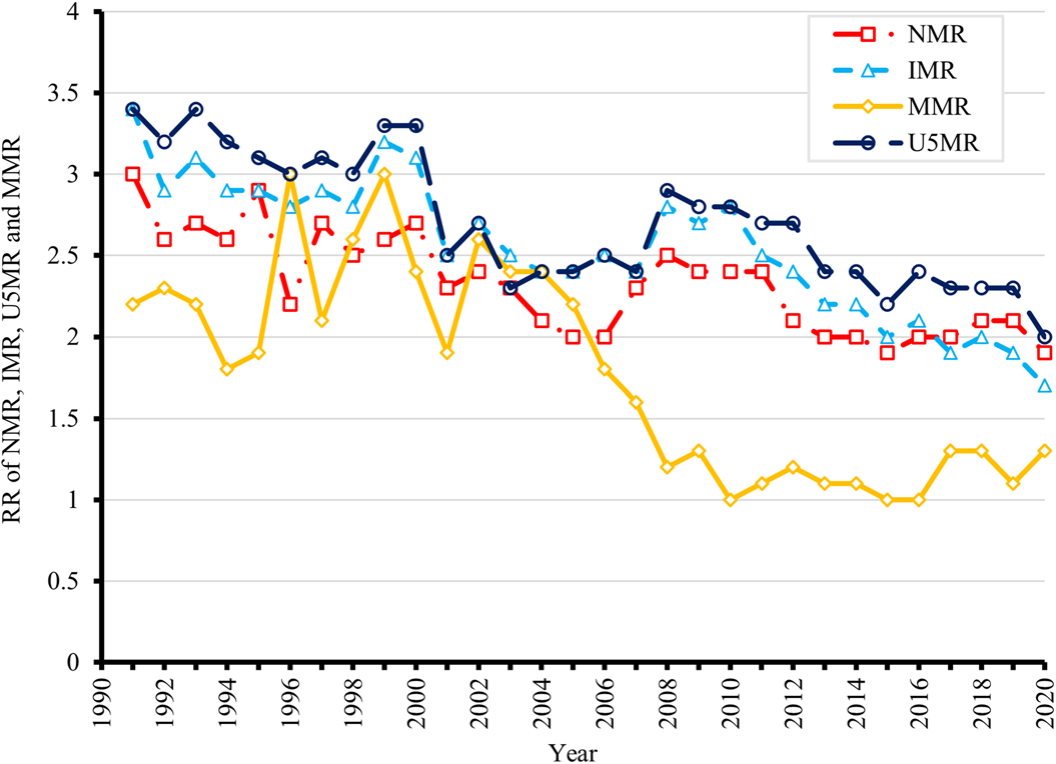
Rate ratios (RRs) of national and urban/rural neonatal mortality rates (NMRs), infant mortality rates (IMRs), under-five mortality rates (U5MRs), and maternal mortality rates (MMRs) in China, 1991–2020.

During the study period, the RDs of NMR, IMR, and U5MR gradually decreased (Fig. 2, Supplementary Table S2). In addition, RDs of MMR gradually diminished from 53.7 per 100,000 live births in 1991 to 0.4 per 100,000 live births in 2010, fluctuated between 2011 and 2020 (ranging from 0.4 to 4.5 per 100,000 live births); the rural/urban ratio of MMR decreased from 2.2 in 1991 to 1.0 in 2010, remained relatively stable between 2011 and 2020 (ranging from 1.0 to 1.3) (Fig. 3, Supplementary Table S2).

### Trends in national child health indicators in China from 1991 to 2020

The results of the joinpoint regression models revealed that the AAPCs of the national NMR, IMR, and U5MR from 1991 to 2020 decreased significantly by around 7–8% (Supplementary Table S3). The AAPCs of the RDs also decreased significantly by around 8% for the three indicators, while the RRs also decreased but more moderately (around 1–2%) (Supplementary Table S4).

### Trends in urban/rural child health indicators in China from 1991 to 2020

**Figure 4:**
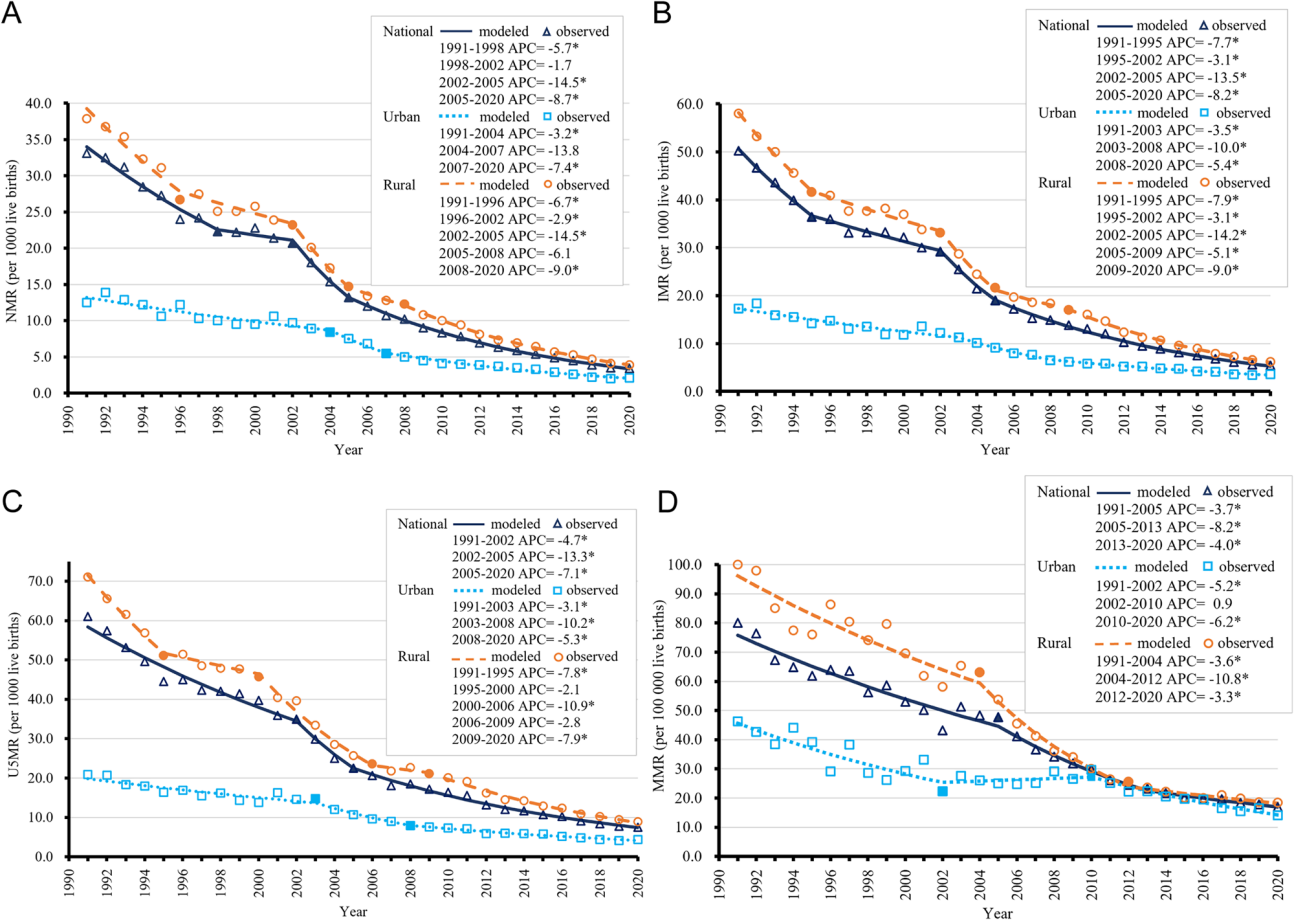
Trends in national, urban, and rural neonatal mortality rates (NMRs) (**A**), infant mortality rates (IMRs) (**B**), under-five mortality rates (U5MRs) (**C**), and maternal mortality rates (MMRs) (**D**) in China from 1991 to 2020, based on joinpoint regression models. Solid symbols indicate statistically significant inflection points. APC, annual percentage change. *P < 0.05.

**Figure 5:**
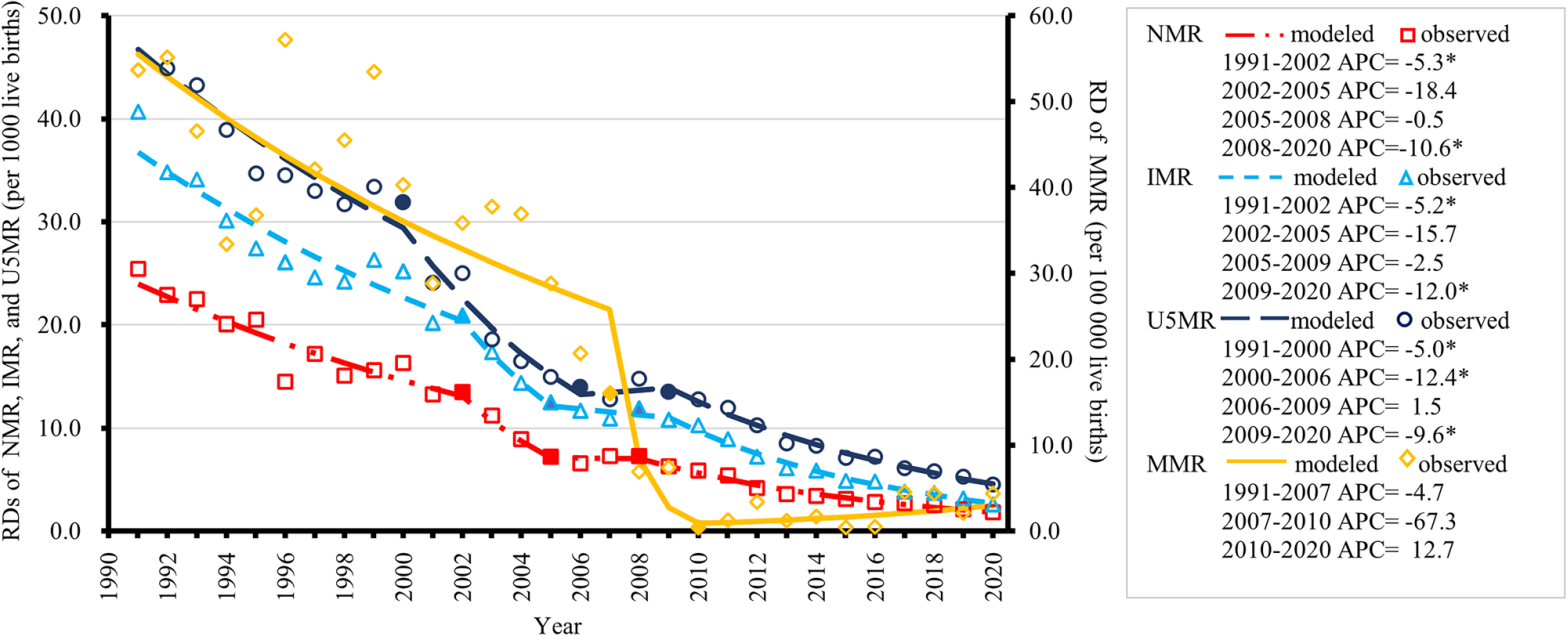
Trends in rate difference (between rural and urban areas, RD) of neonatal mortality rates (NMRs), infant mortality rates (IMRs), under-five mortality rates (U5MRs), and maternal mortality rates (MMRs) in China from 1991 to 2020, based on joinpoint regression models. Solid symbols refer to statistically significant joinpoints. APC, annual percentage change. *P < 0.05.

**Figure 6:**
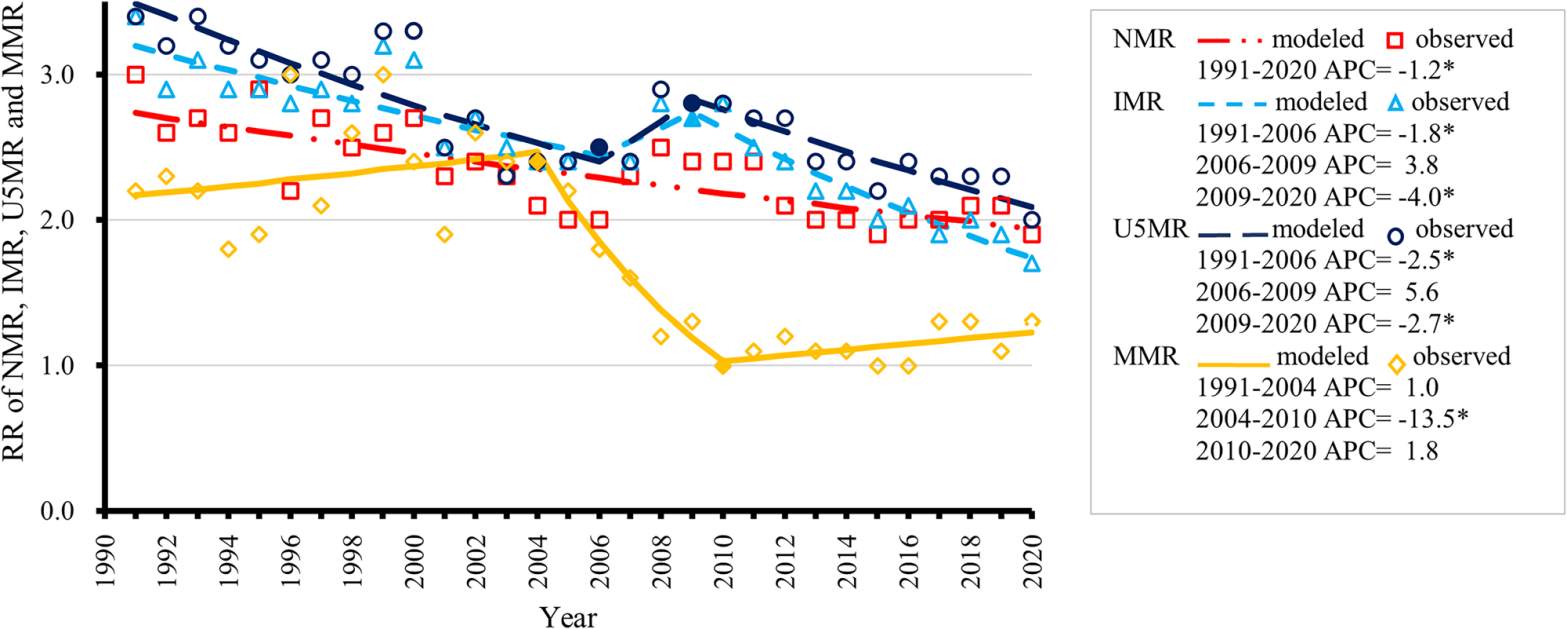
Trends in rate ratio (rural vs. urban, RR) of neonatal mortality rates (NMRs), infant mortality rates (IMRs), under-five mortality rates (U5MRs), and maternal mortality rates (MMRs) in China from 1991 to 2020 by joinpoint regression models. Solid symbols refer to statistically significant joinpoints. APC, annual percentage change. *P < 0.05.

The variation in NMR in urban areas showed three phases: 1991–2004, 2004–2007, and 2007–2020; the variation in rural areas showed five phases: 1991–1996, 1996–2002, 2002–2005, 2005–2008, and 2008–2020 (Fig. 4A, Supplementary Table S3). The RDs in NMR declined from 1991 to 2002 and from 2008 to 2020. We also observed non-significant downward trends from 2002 to 2005 and from 2005 to 2008 (Fig. 5). The RRs of NMR decreased from 1991 to 2020 with no joinpoint (Fig. 6).

The variation in IMR in urban areas showed three phases: 1991–2003, 2003–2008, and 2008–2020; the variation in rural areas showed five phases: 1991–1995, 1995–2002, 2002–2005, 2005–2009, and 2009–2020. The decline in the IMR was greater in rural areas than in urban areas (Fig. 4B, Supplementary Table S3). The RDs in IMR decreased from 1991 to 2002 and then from 2009 to 2020. We also observed non-significant downward trends from 2002 to 2005 and from 2005 to 2009 (Fig. 5). The RRs of IMR decreased from 1991 to 2006 and from 2009 to 2020, while a non-significant upward trend was observed from 2006 to 2009 (Fig. 6).

The variation in U5MR in urban areas showed three phases: 1991–2003, 2003–2008, and 2008–2020; the variation in rural areas showed five phases: 1991–1995, 1995–2000, 2000–2006, 2006–2009, and 2009–2020. The decline was greater in rural areas than in urban areas (Fig. 4C, Supplementary Table S3). The RDs of U5MR declined from 1991 to 2000, from 2000 to 2006, and from 2009 to 2020, while there was a non-significant upward trend from 2006 to 2009 (Fig. 5). The RRs of U5MR decreased from 1991 to 2006, then increased from 2006 to 2009, and decreased again from 2009 to 2020 (Fig. 6).

### Trends in national MMR in China from 1991 to 2020

The results of the joinpoint regression model revealed that the AAPC in national MMR from 1991 to 2020 was − 5.0% (95% CI − 5.7% to − 4.4%) (Supplementary Table S3). During the observation period, the AAPC of the RD was − 9.6% (95% CI − 23.5% to 6.9%), while that of the RR was − 1.9% (95% CI − 4.0% to 0.2%) (Supplementary Table S4).

### Trends in urban/rural MMR in China from 1991 to 2020

The variation of MMR in urban areas showed three phases: 1991–2002, 2002–2010, and 2010–2020; the variation of rural areas showed three phases: 1991–2004, 2004–2012, and 2012–2020. The decline in this indicator was greater in rural areas than in urban areas (Fig. 4D, Supplementary Table S3). The RDs of the MMR showed decreasing trends from 1991 to 2007 and from 2007 to 2010, then an increasing trend from 2010 to 2020, but these changes were not significant (Fig. 5). The RRs of MMR showed increasing trends from 1991 to 2004 and from 2010 to 2020, with a decreasing trend from 2004 to 2010, although the changes were not significant (Fig. 6).

## Discussion

In this study, we analysed trends in MCH indicators and disparities between rural and urban areas in China from 1991 to 2020 using joinpoint regression models. We found that NMRs, IMRs, U5MRs, and MMRs showed overall downward trends at the national level and separately in urban and rural areas. Inequalities between urban and rural areas narrowed considerably over the 30-year period.

The remarkable achievements in MCH in China may be attributed to the rapid socioeconomic development since China's reform and opening-up, the significant improvement in MCH medical services, and effective government policies dedicated to MCH^1,26^. Consequently, the following discussion focused on exploring the potential relationship between the implementation of China's MCH policies and the trends in MCH indicators as well as the trends in the disparities between urban and rural areas.

For the three child mortalities, inflection points were similar. The urban IMR and U5MR declined rapidly from 2003 to 2008, and the NMR declined from 2004 to 2007. Following the outbreak of severe acute respiratory syndrome (SARS) in 2003, the government increased spending on MCH and improved the National Maternal and Child Mortality Surveillance System^27^. In 2004, the Chinese government launched the "Neonatal Asphyxia Resuscitation Training Program" to reduce newborn deaths by asphyxia^28,29^. These changes may have helped enhance the quality of MCH services and reduce NMR, IMR, and U5MR.

The three urban child mortalities showed similar trends to the national indicators, suggesting the high proportion of child deaths that occur in rural areas across the country^30^. In rural areas, the decreasing trends in these indicators were evident from 1991 to 1995 or 1996, presumably linked to economic development. During this period China experienced the fastest economic growth, promoting strong improvements in living conditions, education and health care, even in rural areas^31–33^. However, the decreasing trends in the rural child mortalities slowed from 1995 or 1996 until 2000 or 2002, which could be attributed to the expanded national U5MR surveillance system. Prior to 1995, the country established two nationwide MCH surveillance networks, MMR and U5MR, covering 81 surveillance sites. After 1995, the integration of MMR, U5MR, and birth defect surveillance created a comprehensive MCH surveillance network, expanding U5MR coverage to 116 surveillance sites^34^. This integration may have enhanced the quality of local MCH surveillance and reduced the underreporting of the three rates in rural areas.

The rural U5MR from 2000 to 2006, the other rural and national child mortalities from 2002 to 2005 all accelerated declines. Presumably, the launch in 1998 and 1999 of the World Bank-funded Health and Childhood’s master and subproject, which provided direct financial assistance to pregnancies^35^, has helped reduce child mortalities in resource-limited areas. Meanwhile, in 2001, the State Council promulgated the China Child Development Program (2001–2010) and the China Women's Development Program (2001–2010)^36^. The programs established guidelines for MCH from 2001 to 2010 and promoted extensive health education activities focusing on reproductive health, which had a profound impact on MCH.

After 2008 or 2009, declines in the rural NMR, IMR, and U5MR accelerated again, causing the rate ratio of IMR and U5MR to fall again after 2009. The rural maternal hospital delivery subsidy program launched in 2008 by the former Ministry of Health may played a role^37^. This program improved the resource-use efficiency in MCH services and further increased the rates of hospital births and successful births. In 2009, “Opinions on Deepening Health System Reform”^38^ proposed continuing the subsidy program for rural maternal hospital deliveries and expanding it to cover all pregnant women and newborns in rural areas^39,40^. Moreover, a Basic Public Health Service Project was implemented to provide the complete cycle of health services for newborns and primary prevention measures such as folic acid supplementation for mothers^41^, thus further reducing the three indicators in rural areas. Nevertheless, rural areas continue to suffer from lagging economies^42^, considerable regional differences in MCH services^43^, irregular MCH staff^44^, and generally inadequate resources for MCH^45^. These considerations highlighted the need to continue and reinforce efforts to promote MCH.

In rural areas, MMR showed a rapid decline from 2004 to 2012. This may have been driven by increased funding by the central government for MCH following the SARS outbreak in 2003 and the implementation of the New Rural Cooperative Medical Scheme. The above measures have reduced out-of-pocket costs of hospital births and propelled the utilization of obstetric health resources^27,29^, thus having a positive impact on reducing MMR. However, after 2012, the decline in the rural MMR slowed down. The trend may be linked to the shift in more frequent causes of maternal death towards factors that are difficult to detect and avoid, for example cardiovascular and other noncommunicable diseases^12,46^. Studies have found that a low level of education and health literacy, as well as the limited availability of MCH resources may also trigger a higher MMR in rural areas^47,48^. These firmly entrenched factors make it difficult to reduce the MMR further.

Our analysis highlights the inequality between urban and rural areas in China: the four MCH indicators were consistently higher in rural areas than in urban areas over the 30-year period. This may be attributed to the fact that rural areas are more likely than urban areas to suffer from economic and educational underdevelopment, limited transportation and a lack of medical personnel and infrastructure^2^. Nevertheless, the declines in the four indicators were greater in rural areas than in urban areas throughout the study period, such that the MCH disparity between urban and rural areas narrowed. This narrowing likely reflects the rapid economic development of China, improvement in general educational level as a result of the nine-year compulsory education system^49^, and the adoption of several specific national policies for rural MCH^50^. Despite these advances in rural MCH, substantial inequality remains between urban and rural areas. Hence, future efforts to improve child health should focus on rural areas. After 2010, the MMR was quite similar between urban and rural areas, but the disparity between the two types of areas may have begun widening since 2016. Thus, efforts to improve maternal health should continue and be reinforced.

To achieve further reductions in NMR, IMR, U5MR and MMR, the following recommendations are made at the national level. First, China must prioritize the enhancement of its MCH surveillance system^51^, ensuring not only robust data collection but also regular analysis of trends in MCH indicators and their potential determinants. Second, technical training and skills assessment should continue for personnel who monitor and evaluate MCH services^52^, which is essential for accurately assessing MCH trends and implementing effective interventions. Third, differentiated MCH policies for urban and rural areas should be developed, taking into account their specific needs and challenges.

The strengths of this study include the following: To our knowledge, this is the first work to use joinpoint regression models to analyse trends in NMRs, IMRs, U5MRs, and MMRs at the national level and separately in urban and rural areas in China. Furthermore, this study is the first to explore the potential association between China's MCH policies and trends in MCH indicators, including urban–rural disparities. Additionally, this study uses the most updated available data to examine trends over a 30-year period from 1991 to 2020.

Our study presents several limitations. First, since our primary data were directly sourced from the "China Statistical Yearbook", our findings might be influenced by factors such as changes in monitoring network coverage and guidelines, and underreporting rates. Second, the joinpoint regression model utilized in our study was primarily a trend analysis method and was not intended for causal analysis. Our study could only offer insights into inflection points based on indirect evidence from other studies, necessitating further research to explore causal relationships between these points and various policies. Additionally, due to the comprehensive analysis conducted using the joinpoint regression model, alternative methods for handling and interpreting trend changes were not explored. Future research will focus on collecting additional data on potential influencing factors to further validate the reasons for trend changes, potentially requiring more extensive data collection and analysis to deepen our understanding of maternal and child health indicator changes.

## Conclusions

Our study shows that NMR, IMR, U5MR, and MMR in China as a whole and separately in urban and rural areas decreased substantially between 1991 and 2020. This may reflect the effectiveness of specific national MCH policies in China. However, the hard-won gains in child health during the 30-year period may have plateaued, and disparities between urban and rural areas persist for all core MCH indicators except MMR. Thus, regular analysis of trends in MCH indicators and prompt adjustment of MCH strategies and measures are recommended.

### Supplementary Information

Supplementary Tables.

## Data Availability

The datasets supporting the conclusions of this article are publicly available from the National Health Commission Statistical Information Center (http://www.stats.gov.cn/sj/ndsj/2021/html/E22-18.jpg). Alternatively, interested individuals may contact the corresponding author to request access to the data supporting the findings of this study.

## Abbreviations

MCH: Maternal and child health
IMR: Infant mortality rate
MMR: Maternal mortality rate
NMR: Neonatal mortality rate
U5MR: Under-five mortality rate
N: National
R: Rural areas
U: Urban areas
RD: Rate difference between rural and urban areas
RR: Rate ratio between rural and urban areas
APC: Annual percent change
AAPC: Average annual percent change
CI: Confidence interval
