# Supplementary material for: Trends in maternal and child health in China and its urban and rural areas from 1991 to 2020: a joinpoint regression model
Source: Sci Rep. 2024 Jun 12;14:13480. doi: 10.1038/s41598-024-63689-2 (PMC11169526; doi:10.1038/s41598-024-63689-2)
Supplement: Supplementary file 1 — Supplementary Tables. [file 41598_2024_63689_MOESM1_ESM.pdf]

SUPPLEMENTARY MATERIALS

Supplementary Table S1. National and urban/rural neonatal mortality rates (NMRs), infant mortality rates (IMRs), under-five mortality rates (U5MRs), and maternal mortality rates (MMRs) in China in 1991 and 2020, and percent changes between the two years.

**Population**	**Indicator**	**1991**	**2020**	**Percentage Change, %**
Nationwide				
	NMR	33.1	3.4	-89.7
	IMR	50.2	5.4	-89.2
	U5R	61.0	7.5	-87.7
	MMR	80.0	16.9	-78.9
Urban areas				
	NMR	12.5	2.1	-83.2
	IMR	17.3	3.6	-79.2
	U5R	20.9	4.4	-78.9
	MMR	46.3	14.1	-69.5
Rural areas				
	NMR	37.9	3.9	-89.7
	IMR	58.0	6.2	-89.3
	U5R	71.1	8.9	-87.5
	MMR	100.0	18.5	-81.5

Abbreviations: IMR, infant mortality rate (per 1000 live births); MMR, maternal mortality rate (per 100 000 live births); NMR, neonatal mortality rate (per 1000 live births); U5MR, under-five mortality rate (per 1000 live births).

Supplementary Table S2. Rate differences (RDs) and rate ratios (RRs) of national and urban/rural neonatal mortality rates (NMRs), infant mortality rates (IMRs), under-five mortality rates (U5MRs), and maternal mortality rates (MMRs) in China, 1991-2020

Year	NMR		IMR		U5MR		MMR	
	RD	RR	RD	RR	RD	RR	RD	RR
1991	25.4	3.0	40.7	3.4	50.2	3.4	53.7	2.2
1992	22.9	2.6	34.8	2.9	44.9	3.2	55.2	2.3
1993	22.5	2.7	34.1	3.1	43.3	3.4	46.6	2.2
1994	20.1	2.6	30.1	2.9	38.9	3.2	33.4	1.8
1995	20.5	2.9	27.4	2.9	34.7	3.1	36.8	1.9
1996	14.5	2.2	26.1	2.8	34.5	3.0	57.2	3.0
1997	17.2	2.7	24.6	2.9	33.0	3.1	42.1	2.1
1998	15.1	2.5	24.2	2.8	31.7	3.0	45.5	2.6
1999	15.6	2.6	26.3	3.2	33.4	3.3	53.5	3.0
2000	16.3	2.7	25.2	3.1	31.9	3.3	40.3	2.4
2001	13.3	2.3	20.2	2.5	24.1	2.5	28.8	1.9
2002	13.5	2.4	20.9	2.7	25.0	2.7	35.9	2.6
2003	11.2	2.3	17.4	2.5	18.6	2.3	37.8	2.4
2004	8.9	2.1	14.4	2.4	16.5	2.4	36.9	2.4
2005	7.2	2.0	12.5	2.4	15.0	2.4	28.8	2.2
2006	6.6	2.0	11.7	2.5	14.0	2.5	20.7	1.8
2007	7.3	2.3	10.9	2.4	12.8	2.4	16.1	1.6
2008	7.3	2.5	11.9	2.8	14.8	2.9	6.9	1.2
2009	6.3	2.4	10.8	2.7	13.5	2.8	7.4	1.3
2010	5.9	2.4	10.3	2.8	12.8	2.8	0.4	1.0
2011	5.4	2.4	8.9	2.5	12.0	2.7	1.3	1.1
2012	4.2	2.1	7.2	2.4	10.3	2.7	3.4	1.2
2013	3.6	2.0	6.1	2.2	8.5	2.4	1.2	1.1
2014	3.4	2.0	5.9	2.2	8.3	2.4	1.7	1.1
2015	3.1	1.9	4.9	2.0	7.1	2.2	0.4	1.0
2016	2.8	2.0	4.8	2.1	7.2	2.4	0.5	1.0
2017	2.7	2.0	3.8	1.9	6.1	2.3	4.5	1.3
2018	2.5	2.1	3.7	2.0	5.8	2.3	4.4	1.3
2019	2.1	2.1	3.2	1.9	5.3	2.3	2.1	1.1
2020	1.8	1.9	2.6	1.7	4.5	2.0	4.4	1.3

Note: RD and RR are calculated from rates per 1000 live births (NMR, IMR, U5MR) or 100,000 live births (MMR). IMR, infant mortality rate; MMR, maternal mortality rate; NMR, neonatal mortality rate; U5MR, under-five mortality rate.

Supplementary Table S3. Trends in national, urban, and rural neonatal mortality rates (NMRs) (A), infant mortality rates (IMRs) (B), under-five mortality rates (U5MRs) (C), and maternal mortality rates (MMRs) (D) in China from 1991 to 2020, based on joinpoint regression models.

Indicators		Trend 1		Trend 2		Trend 3		Trend 4		Trend 5		AAPC, % (95% CI)
		Years	APC, % (95% CI)	Years	APC, % (95% CI)	Years	APC, % (95% CI)	Years	APC, %(95%CI)	Years	APC, % (95% CI)	
NMR	N	1991-1998	-5.7 (-6.7, -4.7)**	1998-2002	-1.7 (-5.5, 2.3)	2002-2005	-14.5 (-21.0, -7.5)**	2005-2020	-8.7 (-9.0, -8.4)**	NA	NA	-7.7 (-8.6, -6.8)**
	U	1991-2004	-3.2 (-4.2, -2.2)**	2004-2007	-13.8 (-29.2, 4.9)	2007-2020	-7.4 (-8.3, -6.4)**	NA	NA	NA	NA	-6.2 (-8.1, -4.3)**
	R	1991-1996	-6.7 (-8.3, -5.0)**	1996-2002	-2.9 (-4.6, -1.1)*	2002-2005	-14.5 (-21.0, -7.5)**	2005-2008	-6.1 (-13.2, 1.7)	2008-2020	-9.0 (-9.5, -8.6)**	-7.7 (-8.7, -6.6)**
IMR	N	1991-1995	-7.7 (-10.1, -5.2)**	1995-2002	-3.1 (-4.5, -1.7)**	2002-2005	-13.5 (-20.4, -5.9)*	2005-2020	-8.2 (-8.5, -7.9)**	NA	NA	-7.5 (-8.4, -6.6)**
	U	1991-2003	-3.5 (-4.4, -2.6)**	2003-2008	-10.0 (-14.3, -5.5)**	2008-2020	-5.4 (-6.3, -4.5)**	NA	NA	NA	NA	-5.5 (-6.3, -4.6)**
	R	1991-1995	-7.9 (-10.3, -5.5)**	1995-2002	-3.1 (-4.5, -1.8)**	2002-2005	-14.2 (-21.0, -6.9)**	2005-2009	-5.1 (-8.9, -1.1)*	2009-2020	-9.0 (-9.5, -8.5)**	-7.5 (-8.5, -6.5)**
U5MR	N	1991-2002	-4.7 (-5.4, -3.9)**	2002-2005	-13.3 (-22.7, -2.7)*	2005-2020	-7.1 (-7.6, -6.7)**	NA	NA	NA	NA	-6.9 (-8.0, -5.8)**
	U	1991-2003	-3.1 (-4.0, -2.1)**	2003-2008	-10.2 (-14.8, -5.5)**	2008-2020	-5.3 (-6.2, -4.4)**	NA	NA	NA	NA	-5.3 (-6.2, -4.3)**
	R	1991-1995	-7.8 (-10.6, -4.9)**	1995-2000	-2.1 (-5.1, 0.9)	2000-2006	-10.9 (-12.8, -8.9)**	2006-2009	-2.8 (-11.8, 7.1)	2009-2020	-7.9 (-8.5, -7.2)**	-7.0 (-8.1, -5.9)**
MMR	N	1991-2005	-3.7 (-4.3, -3.1)**	2005-2013	-8.2 (-9.9, -6.5)**	2013-2020	-4.0 (-5.8, -2.2)**	NA	NA	NA	NA	-5.0 (-5.7, -4.4)**
	U	1991-2002	-5.2 (-6.9, -3.5)**	2002-2010	0.9 (-2.7, 4.5)	2010-2020	-6.2 (-8.2, -4.3)**	NA	NA	NA	NA	-3.9 (-5.2, -2.7)**
	R	1991-2004	-3.6 (-4.5, -2.8)**	2004-2012	-10.8 (-12.8, -8.7)**	2012-2020	-3.3 (-5.1, -1.5)**	NA	NA	NA	NA	-5.6 (-6.4, -4.8)**

Abbreviation: IMR, infant mortality rate; MMR, maternal mortality rate; NMR, neonatal mortality rate; U5MR, under-five mortality rate. R, rural areas; N, national; U, urban areas; APC, annual percentage change; AAPC, average annual percentage change; CI: confidence interval; NA, not applicable. * $P < 0.05$. ** $P \leqslant 0.001$.

Supplementary Table S4 Trends in rate differences (RDs) and rate ratios (RRs) of neonatal mortality rates (NMRs), infant mortality rates (IMRs), under-five mortality rates (U5MRs), and maternal mortality rates (MMRs) in China from 1991 to 2020, based on joinpoint regression models.

Indicator		Trend 1		Trend 2		Trend 3		Trend 4		AAPC, % (95% CI)
		Years	APC, % (95% CI)	Years	APC, % (95% CI)	Years	APC, % (95% CI)	Years	APC, % (95%CI)	
NMR	RD	1991-2002	-5.3 (-6.8, -3.8)**	2002-2005	-18.4 (-35.4, 3.2)	2005-2008	-0.5 (-21.2, 25.8)	2008-2020	-10.6 (-11.8, -9.3)**	-8.5 (-11.4, -5.4)**
	RR	NA	NA	NA	NA	NA	NA	NA	NA	-1.2 (-1.5, -0.8)**
IMR	RD	1991-2002	-5.2 (-6.5, -3.9)**	2002-2005	-15.7 (-31.1, 3.1)	2005-2009	-2.5 (-11.8, 7.9)	2009-2020	-12.0 (-13.2, -10.8) **	-8.6 (-10.8, -6.3)**
	RR	1991-2006	-1.8 (-2.5, -1.0)**	2006-2009	3.8 (-13.2, 24.3)	2009-2020	-4.0 (-5.2, -2.9)**	NA	NA	-2.1 (-3.9, -0.3)*
U5MR	RD	1991-2000	-5.0 (-6.5, -3.5)**	2000-2006	-12.4 (-15.8, -8.9)**	2006-2009	1.5 (-14.6, 20.8)	2009-2020	-9.6 (-10.6, -8.5)**	-7.7 (-9.5, -5.9)**
	RR	1991-2006	-2.5 (-3.3, -1.6)**	2006-2009	5.6 (-13.6, 29.1)	2009-2020	-2.7 (-4.0, -1.4)*	NA	NA	-1.7 (-3.8, 0.3)
MMR	RD	1991-2007	-4.7 (-10.4, 1.4)	2007-2010	-67.3 (-93.5, 65.6)	2010-2020	12.7 (-0.7, 27.9)	NA	NA	-9.6 (-23.5, 6.9)
	RR	1991-2004	1.0 (-1.1, 3.1)	2004-2010	-13.5 (-20.8, -5.5)*	2010-2020	1.8 (-1.3, 4.9)	NA	NA	-1.9 (-4.0, 0.2)

Abbreviation: RD, rate difference between rural and urban areas (see Methods); RR, rate ratio between rural and urban areas (see Methods); IMR, infant mortality rate; MMR, maternal mortality rate; NMR, neonatal mortality rate; U5MR, under-five mortality rate. N, national; R, rural areas; U, urban areas; APC, annual percentage change; AAPC, average annual percentage change; CI: confidence interval; NA, not applicable. * $P < 0.05$. ** $P \leqslant 0.001$.
